# The Impact of Stem/Progenitor Cells on Lymphangiogenesis in Vascular Disease

**DOI:** 10.3390/cells11244056

**Published:** 2022-12-15

**Authors:** Rong Mou, Kai Chen, Pengwei Zhu, Qingbo Xu, Liang Ma

**Affiliations:** 1 Department of Cardiology, The First Affiliated Hospital, Zhejiang University School of Medicine, Hangzhou 310003, China; 2 Department of Cardiovascular Surgery, The First Affiliated Hospital, Zhejiang University School of Medicine, Hangzhou 310003, China

**Keywords:** stem cells, progenitors, lymphangiogenesis, lymphavasculogenesis, arteriosclerosis

## Abstract

Lymphatic vessels, as the main tube network of fluid drainage and leukocyte transfer, are responsible for the maintenance of homeostasis and pathological repairment. Recently, by using genetic lineage tracing and single-cell RNA sequencing techniques, significant cognitive progress has been made about the impact of stem/progenitor cells during lymphangiogenesis. In the embryonic stage, the lymphatic network is primarily formed through self-proliferation and polarized-sprouting from the lymph sacs. However, the assembly of lymphatic stem/progenitor cells also guarantees the sustained growth of lymphvasculogenesis to obtain the entire function. In addition, there are abundant sources of stem/progenitor cells in postnatal tissues, including circulating progenitors, mesenchymal stem cells, and adipose tissue stem cells, which can directly differentiate into lymphatic endothelial cells and participate in lymphangiogenesis. Specifically, recent reports indicated a novel function of lymphangiogenesis in transplant arteriosclerosis and atherosclerosis. In the present review, we summarized the latest evidence about the diversity and incorporation of stem/progenitor cells in lymphatic vasculature during both the embryonic and postnatal stages, with emphasis on the impact of lymphangiogenesis in the development of vascular diseases to provide a rational guidance for future research.

## 1. Introduction

The lymphatic vasculature, a complicated blind-ended structure, is responsible for draining the extra interstitial liquid back to the blood circulation, mediating lymphocyte activation, and participating in immune responses [1]. Although most organs/tissues contain lymphatics, so far, no conventional lymphatic vasculature has been identified within the brain (with the exception of meninges) [2,3], bone marrow [4], the posterior part of the eye (retina/choroid/lens/vitreous body) [5,6], and the renal medulla [7,8]. Since the recycling fluid absorbed by lymphatic vessels is ultimately collected into the venous system, the lymphatic vessel system is also considered an important complement to the venous system. Except for the conventional regulation mentioned above, the lymphatic system also takes charge of lipid absorption, immune control, and reverse cholesterol transportation. Importantly, defects in lymphatic function can result in tissue edema and impaired immune responses. With increasing evidence declaring that lymphangiogenesis is closely associated with the prognosis of tissue injury, tumor metastasis, and organ transplantation, it gradually attracted the broad attention of researchers.

Ever since Sabin [9] first proposed that the lymphatic vasculature was derived from veins using an embryo ink injection experiment, an intense debate about the existence of stem/progenitor cells in lymphangiogenesis has continued for over a hundred years. Until recently, the origins of lymphangiogenesis could be concluded into three ways, including originating from the cardinal vein, differentiating from lymphatic stem/progenitor cells, and transdifferentiating from existing blood vessels. Except for the classical venous-derived generation, cells that possess potential differentiation capability, such as hematopoietic cells [10], lymphatic endothelial progenitor cells [11,12], mesenchymal stem cells [13], myeloid lineage cells [14], and adipose-derived stem cells [15], are underestimated. These cells not only appear during the embryonic stage, but also play a significant role in the postnatal lymphangiogenesis in response to various stimuli. For this reason, the exploration of the application of lymphatic stem/progenitor cells to affect the disease process becomes instructive. In the present review, we aim to summarize the latest advances in the research of stem/progenitor cells involved with lymphatic vasculature generation in both the embryonic and prenatal stages and declare the different characteristics of lymphangiogenesis in vascular disease, trying to provide rational inspirations for future research on stem cell therapy

## 2. Progenitor Cells in Embryonic Lymphatic Vasculature Generation

The formation of embryonic lymphvasculogenesis theory could date back to 1902, when Sabin first observed the primitive lymph sacs bud from the pre-existing veins, which initially brings the opinion that venous endothelium was an indispensable source for lymphatic endothelium [9]. However, early descriptive studies by Huntington and McClure [16] suggested that the lymphatic vascular system could develop from mesenchymal lymphangioblasts without any contribution from embryonic veins, which was the first opposing viewpoint. Thereout, whether the lymphatic progenitor cells exist throughout a prenatal organism lifetime soon raised lengthy controversial discussions. Interestingly, in the 2010s there appeared numerous studies that validated Sabin’s vein-derived hypothesis and insisted a solely venous-derived mammalian lymphatic system [17]. But at the same time, people began to realize that there was a huge distinction between embryonic lymphatic origins across species. A collection of studies, which widely involved different species, from the non-mammalian such as birds [18], Xenopus [19], and zebrafish [20,21], to the mammalian-like mouse [17,22,23,24,25], successfully substantiated the dual origins of lymphvasculogenesis in embryos. Admittedly, previous experiments largely relied on skilled and meticulous observation. However, in the last decade, attributed to multiple advanced techniques, including high-resolution microscopy, genetic cell lineage tracing, and single-cell RNA sequencing, more solid data have been obtained and have directly replenished the evidence about the involvement of stem/progenitor cells in lymphatic vasculature generation. Here, we are going to discuss precursor cells and the different origins of embryonic lymphatic vasculature generation.

### 2.1. Embryonic Lymphangiogenesis (Physiological Process)

After many years of research, it has been concluded that nearly all embryonic mammalian lymphatic endothelial cells (LECs) are venous-derived, while some organs and tissues possess diverse non-venous-derived sources in the later embryonic period. Specifically, later than the formation of the vascular system, which is established at around embryo day (E) 9.5, the first lymphatic progenitor cells, Prox1-positive cells, emerge as a polarized cell population within the dorso-lateral aspect of the cardinal and intersomatic veins [25,26]. Importantly, these Prox1-expressing cells are viewed as crucial regulators to specify LEC fate. Compared with previous bipotent endothelial cells in veins, the expression of mature vein endothelial markers, such as CD34 and vWVF, is downregulated in Prox1-expressing cells [27]. Nearly at the same time, LYVE1 is expressed in the venous-derived lymphatic endothelium, which represents the first symbol of the beginning of lymphvasculogenesis. Through a step-wise process, these lymphatic progenitor cells bud and balloon from veins and then migrate and reorganize into lumenal structures to construct the primary lymph sacs [25,26,28]. The formation of primitive lymph sacs is considered as an initial step to the anatomical change in lymphvasculogenesis. Interestingly, Oliver [27] summarized a working model of lymphatic vasculature development and pinpointed that the increased expression of LEC markers would in turn irreversibly propel cell fate toward the lymphatic pathway. Furthermore, endothelial markers are different from organs and tissues, and a review of organ-specific lymphatic vasculature was published as a good reference to acknowledge [29]. Upon terminal differentiation, mature LECs permanently expressed specific cell-type characteristic markers including LYVE-1, Prox-1, and Podoplanin [30]. Prox1 is a transcription factor that is involved in various developmental processes such as cell-fate determination and progenitor cell regulation in a number of organs, plays a critical role in embryonic development, and functions as a key regulatory protein in lymphangiogenesis. Podoplanin is a transmembrane glycoprotein that plays a role in blood and lymphatic vessels separation by triggering C-type lectin domain family 1 member B activation in platelets, leading to platelet activation and aggregation [31,32,33,34,35]. By binding with lactose agglutinin 8, Podoplanin may also participate in the connection of the lymphatic endothelium to the surrounding extracellular matrix. LYVE1 is identified as a major receptor for extracellular matrix glycosaminoglycan hyaluronan on the lymph vessel wall. LYVE1 plays a role in transporting hyaluronan into lymphatic endothelial cells for catabolism and into the lumen of afferent lymphatic vessels reversely as well [36]. It facilitates leukocyte adhesion and migration through lymphatic endothelium by binds to pericelluar hyaluronan matrices [37]. VEGFR3 is a tyrosine kinase receptor for vascular endothelial growth factors VEGF-C and D, which appear to play a role in lymphangiogenesis and the maintenance of the lymphatic endothelium.

**Figure 1:**
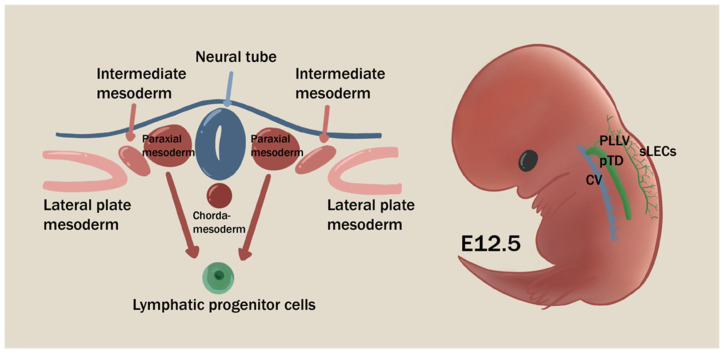
The origin of lymphatic progenitor cells and physiological lymphagiogenesis process at E12.5. Paraxial mesoderm is considered as the major source of lymphatic endothelium at E12.5. The accumulation of initial LECs first sprouts from cardinal vein (CV), then extends dorsally and lumenizes as primordial thoracic duct (pTD) and peripheral longitudinal lymphatic vessels (PLLVs). pTD is connected with the cranial end of PLLVs and develops towards the anatomically mature thoracic duct. PLLVs further branch off as a smaller structure of continuous LEC layer (sLECs).

There was a previous hypothesis indicating that lymph sacs ultimately transform into lymph nodes, which was refuted by Vondenhoff et al. [38], who believed that the initiation of mammalian lymph node formation and the accumulation of initial clusters of lymphoid-tissue-inducer cells did not require lymph sacs. This swarming behaviour of lymphoid-tissue-inducer cells is considered as a vital step to form ordered lymphoid structure as well as a consequence of a self-organizing system [39]. After egress from the cardinal vein, the shape of those Prox1^+^ cells changed from round to oblong until E10.5. At around E12.5, lymphoid-tissue-inducer cells initially cluster to start lymph-node formation [40], which seems to be independent action due to the absence of lymphatic vessels formation nearby. After the initial organization of this process, lymphoid-tissue-inducer cells could be further attracted by CCL21 expressed by LECs [40]. Following the first interactions of lymphoid-tissue-inducer cells with lymphoid-tissue-organizer cells, the induction of VEGF-C expression by lymphoid-tissue-organizer cells is induced and subsequently mediates LECs approach to the lymphatic sacs [41]. The lymphatic node will be completely formed on the seventh day after birth. Moreover, there is a rapid accumulation of Prox1^+^ cells, which extend dorsally to form the peripheral longitudinal lymphatic vessel and further a continuous structure, superficial LECs. Simultaneously, another larger luminal structure, which is closer to the cardinal vein, the primordial thoracic duct, gradually appears. It is connected via a bow-shaped structure with the peripheral longitudinal lymphatic vessel towards its cranial end and develops anatomically in a position corresponding to that of the mature thoracic duct [42]. Finally, the lymphatic network is formed through self-proliferation and polarized-sprouting from the lymph sacs and undergoes sustained growth, remodeling, and maturation to obtain the entire function (Figure 1). Lymph flows into the collecting lymphatic vessels after gathering in the lymphatic capillaries. The collecting lymphatic vessels of the entire body finally converge into two big trunks, that is, the thoracic duct and the right lymphatic duct. The lymph finally enters the blood circulation at the junction of the subclavian veins and the internal jugular veins on both sides.

### 2.2. Venous-Derived Lymphatic Progenitor Cells

Around E9.5, endothelial cells in the dorsal wall of the cardinal vein are the earliest source of Prox1-expressing LEC progenitors [17,25,26]. Prox1-expressing cells, the regulators to determine LEC destiny, separate themselves from the cardinal vein and then bud into stroma in response to VEGF-C/VEGFR3 signaling. While most Prox1-expressing lymphatic progenitor cells will eventually vacate the veins, there are still a few of them that stay and become part of the lymphovenous valves [23]. Acknowledging the cornerstone role of the Prox1^+^ subpopulation of endothelial cells in lymphatic vasculogenesis [25], researchers naturally found a deficiency of Prox1 expression in LEC progenitors among venous endothelial cells in the anterior cardinal veins. Expectedly, the budding and sprouting of the lymphatic system was vanished in Prox1 null mice but the vascular system remained unaffected. So many investigators tend to use the conditional knockout of Prox1 using Cre^ER^/Cre^ERT2^ mice to discuss the relationship between the LEC progenitor and specific source [10,43].

Utilizing different lineage-tracing animals is the basic strategy to validate the hypothesis of venous origin. For instance, a receptor tyrosine kinase expressed in blood endothelial cells and hematopoietic cells, Tie2, is the most adopt marker to trace the venous origin. Cardiac lymphatics have been reported to appear at E12.5, shortly after the development of the coronary vasculature, and integrally formed the complete lymphatic network by P15 [10,44]. Scientists used venous specific Tie2-Cre^ER^, Apj-Cre^ER^, endothelial-specific Cre lines Sox18-Cre^ERT2^, and Cdh5-Cre^ERT2^ mice to prove that the majority of cardiac lymphatics were derived from veins [45,46,47]. However, there were still a few lymphatic vessels that were not labeled, in consideration of the high recombination rate of these two drivers, and it implied that the possibility of non-venous origin remained existing. As a solution, Pdgfrb-Cre for labeling mural cells, including the vascular smooth muscle cells, pericytes, and hepatic stellate cells, was used and succeeded in tracking embryo LECs in the heart and found evidence about the non-venous-derived origins [10,48]. To summarize, while the non-venous origins show the heterogeneity of lymphatic progenitor cells, the venous-derived origin is still predominant in multiple organs.

### 2.3. Non-Venous-Derived Lymphatic Progenitor Cells

Among the lineage-tracing strategies of mouse embryos, Runx1-expressing cells, labeling the hematopoietic components, are shown to not be involved in the lymph sacs formation or lymphatic vasculature [17]. Similarly, by using Kit-Cre^ERT2^ and Gata2-deficient embryos, Pichol-Thievend et al. [47] excluded the hematogenic endothelium as a source of LEC progenitors in embryonic mouse skin. However, while Martinez-Corral et al. [22] found the comparable performance in dermal LECs through tracing Tie2-positive endothelial/hematopoietic cells and Vav-definitive hematopoietic cells, they surprisingly found that part of the mesenteric lymphatic vasculature was from c-Kit lineage progenitor cells, which were conventionally regarded as the hemogenic endothelial origin [48]. It was worth noting that the dual origin of mesenteric lymphatic vessels was declared by Mahadevan et al., that there exists a separate population of lymphatic progenitors that forms mesenteric lymphatic vasculature, which is dependent on the expression of Pitx2 (paired-liked homodomain transcription factor-2) [49]. These findings provided solid evidence, indicating that the differentiation potential of non-venous-derived lymphatic progenitor cells is not homogenous in different organs or tissues. In other words, the therapeutic use for lymphatic vasculature regeneration should characterize in consideration of the corresponding cellular sources of progenitors, which may not be useful. Analogously, the lymphatic vasculature of embryonic mouse heart was proven to be derived from both extra-cardiac venous endothelium and lymphatic endothelial progenitors in yolk sac haemogenic endothelium [10]. The initial negative results about hematopoietic cells may be explained by the oversight of organ specificity and an incomplete yolk sac, including aggregations and primitive hematopoietic derivatives labeling. More precisely, Klotz et al. [10] confirmed the contribution of the yolk sac by Vav1-Cre; R26-tdTomato mice, therefore, ensured Vav1 hemogenic lineage contributed to cardiac LECs as a non-venous origin. However, the contribution of hematopoietic cells integrating into lymphatic vasculature is very small and the dysplasia of cardiac lymphatic vessels during the Tie2-Cre; Prox1^fl/fl^ mutants’ embryo period, is recoverable after birth.

Moreover, using Csf1r-iCre; Z/EG (lacZ/EGFP) and LysMCre; ROSA26R mice to label myeloid lineage cells, Gordon, E.J., et al. [50] excluded macrophages as a reservoir of lymphatic endothelial progenitor cells in both the mouse embryo and the tumor microenvironment. Though they detected myeloid LYVE1^+^ macrophages and seemed to locally integrate into lymphatic vessels, the absence of a crucial marker of lymphatic endothelial cell identity PROX1 explained that they are just undergoing the intimate association but not the identity transformation [10]. The above evidence additionally emphasized that the detection of important LEC markers was necessary to distinguish the facticity of transdifferentiation. Hence, the previous statement about lymphatic endothelial progenitors in yolk sac haemogenic endothelia is being queried. Lineage-tracing analysis in transplantation studies revealed that PAX3^+^ [51] (arising embryonic myoblasts and myofibers), VEGFR2^+^ (highlighting hematopoietic, vascular, and muscle cells) [52], and Myf5^+^ (initiating muscle differentiation) somitic cells [53] are able to differentiate into endothelial cells as bipotent precursors for the skeletal muscle and endothelium of the limb, while the source of Prox1^+^ lymphatic progenitors and endothelial cells in the dorso-lateral wall of the E9.5 cardinal vein is considered to be derived from the Pax3 lineage [53]. Additionally, the second heart field, a multipotent cell population managing heart morphogenesis, labeled by Isl1-expressing progenitors in the pharyngeal region, also serves as a source of LECs [43]. In conclusion, improved lineage-tracing techniques and rigor definition for LEC differentiation are conducive to the recognition of non-venous-derived lymphatic progenitor cells.

## 3. Stem/Progenitor Cells in Postnatal Lymphangiogenesis

Under physiological conditions, lymphangiogenesis does not only exist in the embryo stage, but also happens in the postnatal period. To illustrate, Schlemm’s canal, lined by endothelial cells expressing LEC markers (PROX1, VEGFR3, integrin α9) [54,55,56,57], is a lymphatic intermediate vessel with the function of draining aqueous humor [29]. The Schlemm’s canal is originated from transscleral veins that are derived from episcleral and choroidal vessel plexus [54,58] and acquired lymphatic phenotypes through upregulating PROX1 [56]. Subsequently, Tie2 is expressed in the PROX1^+^ Schlemm’s canal endothelial cells and maintained at a high level to critically regulate integrity during adulthood [59]. VEGF-C/VEGFR3 signaling also plays a crucial role in the development of Schlemm’s canal, delivering VEGF-C into adult eyes leading to the sprouting, proliferation, and growth of Schlemm’s canal [54]. For another example, intestinal lacteal, a lymphatic capillary in the intestinal villi, plays an important role in absorbing nutrients and tracking immune cells into mesenteric lymph nodes [60]. Contrasted with the majority of adults lymphatic vessels, which are quiescent, the intestinal lacteals show permanent regeneration under physiological conditions [61,62]. In 2015, Bernier-Latmani et al. demonstrated that the activation of VEGFR3 and VEGFR2 leads to the expression of DLL4 and thus activates the NOTCH signaling, inducing the proliferation of intestinal lacteal [61].

In contrast to the physiological process of lymphangiogenesis after birth, it seems that people are keen to pay attention to pathological conditions. As evidenced in chronic inflammation [63], acute injury [64], tumorigenesis [65,66], and organ transplantation [67], the presence of lymphangiogenesis is considered as a critical event mediating liquid drainage and immune reactions during the postnatal period. Instead of sprouting, remodeling, and maturation from pre-existing lymphatic vessels, lymphangiogensis is also dependent on the contribution of stem/progenitor cells. Similar to research on the developing embryo, advancements in the lineage tracing and specific markers for lymphatic endothelium have allowed us to better figure out the mechanism and cell fate of lymphatic progenitor cells during postnatal lymphangiogenesis. By checking the expression of classical lymphatic endothelial cell markers, such as Prox-1, Podoplanin, Lyve-1, VEGFR2, and VEGFR3, we tend to capture the cells that have the potential to differentiate into LECs as well as representing a high-proliferative feature. Nonetheless, distinct from the venous/non-venous origin of embryonic lymphangiogenesis, a great individuation of postnatal LEC progenitors, which locate throughout different parts of bodies, has been embodied according to specific disease type. Herein, we introduce the main sources of stem/progenitor cells during postnatal lymphangiogenesis, discussing the biology of these cells and how they mediate or benefit the diseases.

### 3.1. Lymphatic Endothelial Progenitor Cells

Lymphatic endothelial progenitor cells (LEPCs) have been recognized as main contributors to developmental and postnatal lymphangiogenesis [11,12]. The broad participations of LEPCs are declared in injury, inflammation, tumor, and transplantation [68,69]. As an important source of LEPCs, the isolation of peripheral blood cells can successfully acquire two distinguishable circulating progenitors, including classical endothelial progenitors (high-level expression of VEGFR-1 but low-level VEGFR-3) and LEPCs (high-level expression of PROX-1, VEGFR-3, Podoplanin, and LYVE-1 but low-level VEGFR-1) [70]. In addition, the existence of an analogous group (VEGFR3^+^/Podoplanin^+^/CD11b^+^ LEPCs) has also been confirmed in human umbilical cord blood [71]. 

For the application, the impairment of lymphatic drainage generally occurs in cardiovascular diseases and is commonly viewed as an aggravating factor to exacerbate cardiac edema, inflammation, fibrosis, and arrhythmia [72,73]. Since CD34^+^VEGFR3^+^PROX1^+^CD45^−^CD11b^−^CD68^−^ LEPCs isolated from bone marrow have been reported to possess a great potential to differentiate towards LECs, injecting or transplanting LEPCs to stimulate lymphangiogenesis becomes a potent therapeutic method [74]. In fact, CD34^+^ VEGFR3^+^ LEPCs isolated from bone marrow were injected into myocardium around the peri-infarct region and consequently transdifferentiated and incorporated into lymphatic vessels, which did not contribute to blood endothelial cells [74]. Admittedly, the LEPC-induced repair process does not always act as a rapid response, so the improvements to promote the survival, retention, and viability of transplanted LEPCs remain a challenge for future therapeutic approaches. Interestingly, a subpopulation in human fetale, labeled with CD34 and VEGFR3 cells, co-expresses the stem cell marker CD133, suggesting the other existence of lymphatic progenitor cells in the human body [75].

### 3.2. Mesenchymal Stem Cells

Mesenchymal stem cells (MSCs) are a subpopulation of stem cells, which are usually isolated from the umbilical cord, bone marrow, and adipose tissue. MSCs are known for their great potential to differentiate into bone, fat, and endothelial cells within multiple tissues [76]. Moreover, MSCs were also sensitive to the lymphangiogenic VEGF-C signal and were inclined to acquire a lymphatic phenotype both in vivo and in vitro [77]. For instance, the TGF-β-responsive, VEGFR-3-positive SG-2 MSCs, which retain both osteogenic and adipogenic differentiation potentials, can be induced to differentiate into LECs by VEGF-C stimulation or inhibited by TGF-β signal [13]. In the wounded heart suffering from myocardial infarction, a group of mesenchymal markers, PDGFRα^+^ MSCs with concurrent high expressions of podopanin, Prox-1, and VEGFR-3, manifest a predominant ability to generate lymphatic endothelial cells and impact the outcome of the myocardial repair process [69]. In addition, autologous stem cell therapy usually adopts mesenchymal stem cells as a reliable and suitable source to treat limb ischemia [78]. However, the difficulty and costliness to get sufficient stem cell storage limits the diffusion of application. Herein, people turn to utilize the xenograft of porcine MSCs. Yamada et al. [79] proposed that xenotransplantation of porcine bone marrow MSCs contributed to the improvement of mouse hind limb ischemia through both angiogenesis and especially lymphangiogenesis, showing the potential of being an alternative source for stem cell therapy (Table 1). Nevertheless, a lack of direct evidence that confirmed the incorporation of MSCs into lymphangiogenesis is flawed, which means we cannot rule out the disturbance of the paracrine effect. Actually, when the joint injection of MSCs and endothelial progenitors show cooperative interactions, MSCs are more likely to distribute around lymphatic vessels other than those directly involved in lymphangiogeneis, which represents a negative viewpoint for MSCs-LECs [80].

Despite increasing lymphangiogenesis being beneficial in lymphedema after injury, the swelling due to lymph accumulation in the extracellular space, it does not always acquire same compliment especially when involved with tumor prognosis and organ transplantations. In other words, lymphangiogenesis can be a double-edged sword, which can be evidently embodied in the application of MSCs. The hypoxia microenvironment caused by tumorigenesis or transplantation surgeries tends to attract and stimulate the MSCs accumulation. On this aspect, the following proliferation of lymphangiogenesis by MSC differentiation and the remodeling of existing lymphatics are thought to be the initial events in cancer metastasis and inducing immunological rejection. These consequences make the generation of lymphatics a harmful biological behavior [65,66]. Hence, it can be predicted that the suppressing of the VEGF-C/VEGFR3 pathway will decrease the lymphatic fluid drainage in cardiac and cornea allografts, reduce the negative effect of both innate and adaptive immunity, and therefore prolonged the survival time of grafted organs [90,91]. In this situation, the specific-disease-associated mechanisms have warned MSCs induced the generation of lymphangiogenesis, and the therapy should be carefully discussed.

### 3.3. Myeloid Lineage Cells

Myeloid lineage cells have been established to play important roles during both embryonic- and inflammation-stimulated lymphangiogenesis [50,89,92]. Previously, Maruyama et al. [14] reported that macrophages could support the lymphangiogenesis either by transdifferentiation or stimulating preexisting lymphatics, regarding macrophages as a source of VEGF-C to trigger the growth or hyperplasia of lymphatic vessels. Maruyama et al. [14] mentioned that myeloid cells contributed to lymphangiogenesis in the inflamed cornea and had the ability to form lymphatic vessel-like structures in vitro. In fact, different macrophage subtypes have been well defined by specific markers and corresponding lineage-tracing techniques. Among various subtypes, M1/M2 type macrophages were distinguished by the promotive or suppressive effect on the inflammatory responses, and this discrepancy also influences their impact on the lymphangiogenesis [93,94,95,96]. Specifically, in the renal fibrosis microenvironment, M1 macrophages were deemed as stimulative factors and could contribute to the lymphangiogenesis through the classical VEGF-C/VEGFR3 signaling pathway. Furthermore, by promoting M1-macrophage polarization and increasing the LEC expression, M1 macrophages could transdifferentiate into LECs both in vivo and in vitro [97]. Comparatively speaking, M2 macrophages, characterized by eliminating inflammation to promote tissue repairment [98], were considered to have a low inclination to transdifferentiate into LECs, as proved by the low expression of lymphatic endothelium markers [99]. To trace the fate of cells of the myeloid lineage during tumor lymphangiogenesis, Zumsteg et al. [89] transplanted TRAMP-C1 murine prostate cancer cells into CD11b-Cre; Z/EG mice and subsequently detected the LYVE-1, Prox-1, and F4/80 triple-positive cells in tumor lymphatic vessels, testifying an already myeloid-committed hematopoietic lineage origin. In another tumor implantation model, Podoplanin^+^CD11b^+^ cells derived from bone marrow can function as lymphatic progenitor cells and participate in postnatal lymphatic neovascularization through both lymphvasculogenesis and lymphangiogenesis [83]. Whereas, the opposing evidence showed that in adult LysMCre; ROSA26R mice implanted with Lewis lung carcinoma or EL4 lymphoma cells, lymphangiogenesis arose independently of the macrophage lineage, a phenomenon similarly reported in tumor-stimulated lymphangiogenesis [50]. Except for macrophages, monocytes in vitro might also transdifferentiate into either blood vascular endothelial cells or lymphatic endothelial cells, and the LEC phenotype is easier to acquire under an inflammatory environment [100,101,102]. CD14^+^ monocytes induced by the endothelial medium EGM2 are able to express lymphatic endothelial markers Prox-1, VEGFR-3, LYVE-1, Podoplanin, and pan-endothelial markers vWF, CD144, and VEGFR-2 [103], but it has not been confirmed by in vivo models.

### 3.4. Adipose-Tissue-Derived Stem Cells

Lymphedema is a chronic disease, which ultimately causes severe and permanent damage within involved organs. Among the underlying stem cell treatments, adipose-tissue-derived stem cells’ (ADSCs) extraction is known for minimal donor site impairment and less discomfort from patients. Except for the non-invasive harvesting technique, ADSCs also possess other advantages, such as convenient acquisition because they are easily isolated from adipose sediment after digestion, higher proliferative capacity, and stronger genetic and morphologic stability. Therefore, ADSCs are constantly regarded as potential therapeutic targets in various diseases, especially in lymphedema.

In vitro ADSCs are usually defined as positive for CD13, CD29, CD44, CD49d, CD73, CD90, and CD105 and negative for hematopoietic cell markers such as CD14, CD31, CD45, and CD144 [15]. By being injecting with ADSCs, both animals and human generally therefore benefited from lymphedema treatment, showing that ADSC-based therapy is an important key to the future treatment for secondary lymphedema [104,105]. However, the view of this differentiation was only verified in vitro, and it seems more promising to figure out how the paracrine action occurs.

Apart from the direct differentiation ability, the paracrine effect of ADSCs has been elucidated. Compared with the bone-marrow-derived MSCs, ADSCs display more immunomodulatory functions with secreting basic fibroblast growth factor, interferon-γ, insulin-like growth factor-1, VEGF-C, and HGF [106,107]. More importantly, the secreting components would constantly adapt to the changing microenvironment, and this characteristic perfectly fits the continuously changed homeostasis of tumor, ischemia, and tissue injury. For example, in hypoxia, ADSC-derived extracellular vesicles are loaded with decreased exosomal miR-129 expression, which resulted in the upregulation of HMGB1 in LECs and led to AKT activation and lymphangiogenesis enhancement [108]. Another aspect of injection, in addition to exosome secretion, adipose-tissue-derived microvascular fragments, carrying segments of micro blood/lymphatic vessels as well as other stem cells such as endothelial progenitor cells (Sca-1^+^/VEGFR-2^+^) and multipotent mesenchymal stromal cells (CD29^+^, CD44^+^, CD73^+^, CD90^+^, and CD117^+^), have attracted much attention [109]. Although this therapeutic method has not been extensively used, it can really help to differentiate LECs by utilizing the current existing stem cells, but is still waiting for further verification (Table 1). 

## 4. Mechanisms of Differentiation in Lymphatic Progenitor Cells

### 4.1. Embryonic Mechanism

No matter whether in the embryonic or postnatal stage, Prox1 has shown great significance in accumulating in developing LECs progressively over the time posted. In the cardinal vein, where the embryonic lymphatic vessels chiefly arise from the cellular fate of Prox1-positive lymphatic precursor cells, they have asymmetrical division; namely, while one daughter cell progressively upregulates Prox1 and becomes LECs, the other downregulates Prox1 and remains in the vein [110]. Meanwhile, SOX18 and COUP-TFII, as two coordination factors for Prox1, are indispensable when LEC progenitors start to differentiate and acquire the expression profile of LECs [111,112]. Although SOX18 is only transiently expressed in the cardinal vein during LEC fate induction, it triggers the expression of Prox1 in venous lymphangioblasts and determines the development of lymphatic vasculature. Further, it has been reported that MAPK/ERK signaling is an activator of Sox18 in the embryonic veins [113]. ERK activation determines LEC fate specification and the excessive ERK activation can lead to the lymphatic abnormalities observed in Noonan syndrome. In comparison, COUP-TFII activity has been discovered to help maintain the LEC phenotype by direct interaction with Prox1. Prox1 and COUP-TFII synchronously bind to the endogenous cyclin E1 promoter and physically interact during the differentiation and maintenance of lymphatic vessels [114]. 

As mentioned above, activating VEGF-C/VEGFR3 signals through ERK are able to upgrade Prox1 and drive the progenitor differentiation, expansion, and sprouting from the posterior cardinal vein at the earliest stage of lymphatic development [113,115]. On this account, the VEGF-C/VEGFR3/ERK axis is considered as a direct inducer to force G1 cell-cycle arrest and result in enhanced lymphatic sprouting. In the upstream pathway, the mmp13b-par1-gnai2a axis regulates the lymphangiogenic process in the lymphatic trunk of zebrafish by promoting VEGF-C/VEGFR3/ERK1/2 signaling through hematopoietically expressed homeobox, which induces prox1a expression in series [116]. A lack of hematopoietically expressed homeobox may cause a diminished number of lymphatic precursors and a defect in tip cell specification or behavior [117]. In contrast, the inhibitory splicing program, Nova2, can constrain the ERK signaling and further restrict the size of the specified progenitor pool [118]. Additionally, as the downstream for paracrine VEGF-C-activating LEC progenitor, Yap1 is another essential regulator through the VEGFR3/ERK signaling pathway. Specifically, MZyap1^−/−^ mutants fail to form a lymphatic network in the zebrafish trunk. Despite being crucial for lymphangiogenesis, its paralogue Taz can partly compensate the absence of Yap1 [119]. Moreover, the Dll4/NOTCH1 and Dll4/NOTCH4 signaling pathways could regulate the expression of VEGFR3 via control over EphrinB2 expression, modulating the lymphatic development, which was detected both in embryonic and early postnatal dermal lymphatic vasculature. Afterwards, either NOTCH1 and NOTCH4 blocked the VEGF-C activation of Ras/ERK signaling to varying degrees [120].

**Figure 2:**
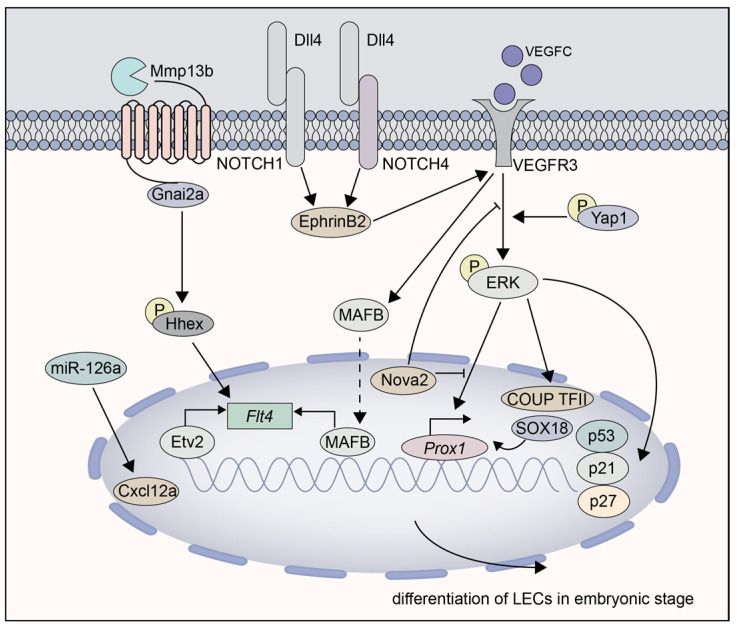
The molecular pathways involved in LEC differentiation from embryonic progenitors. (1) The mmp13b-par1-gnai2a axis activates VEGF-C/VEGFR3/ERK1/2 signaling through HHEX, which induces Prox1 expression and promotes the lymphangiogenesis; (2) Prox1, COUP-TFII, and SOX18 synchronously bind to the endogenous cyclin E1 promoter and interact with the differentiation and maintenance of LEC feature; (3) The expression of p53, p21, and p27 significantly promote the lymphatic sprouting in response to VEGF-C/VEGFR3 stimulation; (4) Dll4/NOTCH1/NOTCH4 axis regulates the expression of VEGFR3 via EphrinB2/Ras/MAPK signaling pathways; (5) The inhibitory splicing program Nova2 and effector Yap1, respectively, suppress or promote VEGFR3/ERK signaling pathway; (6) MAFB or Etv2 directly regulates the Prox1 and Flt4 expression; (7) miR-126a/Cxcl12a and VEGF-C/Flt4 signaling pathways cooperate and jointly direct LECs sprouting and extension.

In the downstream of ERK signaling, the lymphatic sprouting process can also be significantly induced by the expression of p53, p21, and p27 in the dorsal posterior cardinal vein in response to VEGF-C/VEGFR3 signaling stimulation, impelling the autonomous ability to leave the posterior cardinal vein [121]. In addition, transcription factor hematopoietically expressed homeobox is also recognized as a crucial upstream regulator. A lack of this homeobox may cause a diminished number of lymphatic precursors and defected tip cell specification and behavior [117]. Another transcription factor MAFB is declared as a direct target of VEGF-C/VEGFR-3 signaling in LECs, regulating several LEC differentiation genes including Flt4 [111]. In addition, it has been revealed that Etv2 can directly regulate Flt4 expression within the posterior cardinal vein, even prior to the initiation of lymphangiogenesis. Nonetheless, the direct contact between Etv2 and PROX1a was excluded by the researchers [122]. Interestingly, the silencing of miR-126a can also strongly affect the formation of parachordal lymphangioblasts and the thoracic duct in zebrafish embryos [123], demonstrating that the miR-126a/Cxcl12a and VEGF-C/VEGFR3 signaling pathways cooperate and jointly direct lymphatic endothelial cell sprouting and extension (Figure 2).

### 4.2. Adult Mechanism

Different from the embryonic differentiation pathways, the lymphatic progenitor cells in adulthood depend on the specific progenitor type and disease microenvironment. For example, in renal transplantation, VEGF-C is abundantly expressed by the secreting of tubules and interstitial cells. Receiving the signals from the VEGF-C/VEGFR3 pathway, the iNOS expression is first increased in macrophages and the autophagy is suppressed. These changes upregulate the M1 marker expression and create more polarizations. As a result, classically activated (M1) macrophages tend to predominantly differentiate into LECs in vivo and in vitro [99]. Having known about the pluripotential differentiation capacity of ADSCs, Yan et al. [124] implanted ADSCs enfolded with matrigel plugs in mice and explored the impact for differentiation under TGF-β1 and VEGF-C stimuli. The inhibition of TGF-β1 and activation of VEGF-C promote the LEC-specific marker expressions in different levels. Interestingly, the combination use of TGF-β1 and VEGF-C stimuli could fade the positive effect in lymphangiogenesis, suggesting that TGF-β1 may obstruct the VEGF-C signaling pathway [124]. Nonetheless, in a mouse peritonitis model, myeloid and peritoneal macrophages were mobilized around lymphatic vessels and macrophage-derived LEPCs were incorporated into the inflamed lymphatic vasculature. In this condition, it rises NF-κB, p50, and p65 levels and therefore regulates the transcription of VEGFR-3 in both LECs and macrophage-derived LECPs. Meanwhile, inflammation-induced VEGFR-3 simultaneously elevated the expression of several LEC markers including VEGF-C and VEGFR3 and furthered highly upregulated gene expression including Flt4, LYVE1, and PDPN, suggesting an autocrine loop, and it may restrain the LEC-preferred cell fate of LEPCs [88]. In addition, the activation of the TLR4/NF-κB pathway induces myeloid cells to differentiate into M-LEPC like cells, thereby influencing the downstream targets, the VEGFR3/VEGF-C pair [125]. 

**Figure 3:**
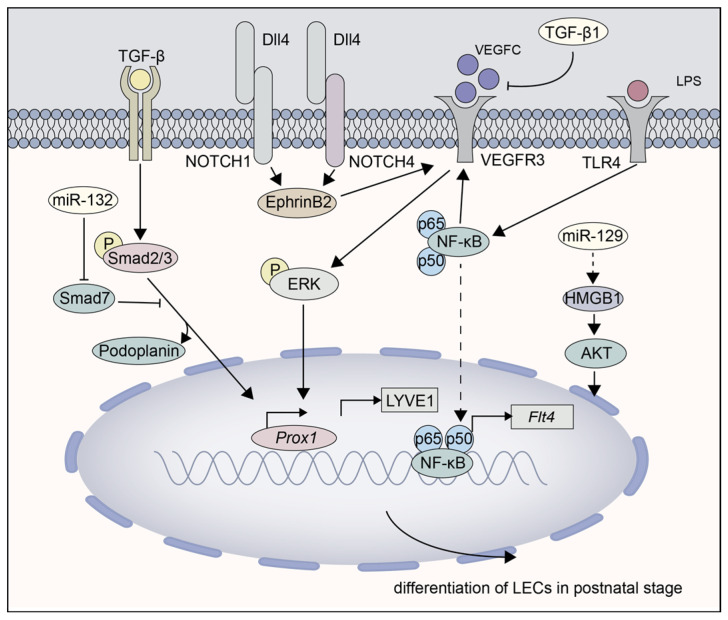
The molecular pathways responsible for LEC differentiation during adulthood stage. (1) The inhibition of TGF-β1 and activation of VEGF-C promote Podoplanin and Prox1 expressions in different levels. (2) NF-κB, p50, and p65 regulate the transcription of VEGFR-3 in both LECs and macrophage-derived LECPs in a mouse peritonitis model. (3) LPS-activation of TLR4/NF-κB pathway influences the downstream targets of the VEGF-C/VEGFR3 pair, inducing myeloid cells to differentiate into M-LECP. (4) VEGF-C-treated adipose-tissue-derived stem cells were proved to promote lymphangiogenic function by regulating TGF-β/Smad signaling. (5) Similar with the embryonic stage, Dll4/NOTCH1/NOTCH4 axis regulates the expression of VEGFR3 via EphrinB2 /Ras/MAPK signaling pathway. (6) miR-129/HMGB1/AKT signaling in adipose-tissue-derived stem cells stimulates the lymphangiogenesis.

Instead, turning to focus on mesenchymal cells, MSCs have been used to treat a variety of diseases due to their anti-inflammatory properties, for instance suppressing pro-angiogenic monocyte and macrophage recruitment. TSG-6 secreted by MSCs can bind to CD44 on macrophages and interfere with TLR2/NF-κB signaling, taking effect by preventing macrophages from differentiating into LECs [126]. Referring to the especial member of MSCs, VEGF-C-treated ADSCs were proved to have the ability to promote lymphangiogenic response by regulating TGF-β/Smad signaling. Moreover, it indicated that miR-132 enhanced the activation of TGF-β signaling, in part by inhibiting the negative regulator smad7 [127]. Nevertheless, extracellular vesicles, hypoxia-conditioned ADSCs, could also induce lymphangiogenesis, which was mediated by miR-129/HMGB1/AKT signaling, illustrating the predominant impact of mesenchymal stem cells, which may be mainly ascribed to paracrine effect [108]. Unlike the conditions of inflammation or injury, the newly formed lymphatic vessels in tumors mostly proliferated from preexisting lymphatics, and the contribution of differentiation by myeloid cells was still under continuous argument [128,129]. So, currently, our understanding of the signaling pathway that regulates the stem/progenitor cell differentiation is illustrated in Figure 3, although it remains limited.

## 5. Lymphangiogenesis in the Development of Vascular Disease

The vascular disease is an indispensable part of cardiovascular diseases. The occurrence and development of diseases such as aneurysm, atherosclerosis, and transplant arteriosclerosis, are closely related to cholesterol deposition, immune inflammatory reaction, and lymph stasis [130,131,132]. During the disease development, lymphatic vessels not only play the role of drainage and excretion, but also transport antigen presenting cells to local lymph nodes to participate in the induction of the immune response. Since the studies about the lymphangiogenesis in vascular diseases remain scarce, the therapeutic target on lymphangiogenesis is still under debate. In this section, we will focus on the lymphangiogenesis in vascular diseases and discuss the future research direction of lymphatic vessels in vascular biology.

### 5.1. Abdominal Aortic Aneurysm

Abdominal aortic aneurysm (AAA), a progressive segmental abdominal aortic dilation, is usually asymptomatic and often diagnosed only after a catastrophic rupture event, which leads to a high mortality rate [133,134]. Inflammatory infiltration and the loss of elastin fibers along the site of the aortic wall are considered crucial features in abdominal aortic aneurysms. In an earlier stage, macrophages invade the forming abdominal aortic aneurysm and therefore significantly accelerate its pathogenesis [135]. In fact, newly generated lymphatic vasculature was reported to be insufficient to clear the fragment of tissue and therefore cause lymph fluid stasis and exacerbate aneurysm disease [136]. Lymphatic vessels play a very important role in abdominal aortic aneurysm and may be responsible for both the drainage of the inflammatory infiltration and regulation of the immune response [137]. Researchers can often observe lymphatic neogenesis in the outer membrane at the lesion, which is generally considered to be a reasonable response to the loss of adventitia lymphatic vessels and lymph stasis. Interestingly, there is no lymphatic vasculature distributed in normal aortic intima and tunica media; however, we can detect a significant increase in the expression of the lymphatic-vessel-specific markers Podoplanin and Prox-1 in the nuclei among lymphangiogenic cells. In addition, the expressions of VEGF-C/D/VEGFR3 and VEGF-A/VEGFR1 signaling were elevated predominantly on the aneurysm vascular wall, which may have resulted from infiltrated macrophages, T cells, B cells, and other inflammatory cells [138,139,140]. The distinctive expression of VEGF-C and MMP-9, major drivers of lymphangiogenesis, in large macrophages, identified its vital contribution in intima/media [138,141]. T cells and neutrophils were revealed to accelerate macrophage recruitment in the region; on the contrary, B cells seem not be involved in lymphangiogensis in abdominal aortic aneurysm walls due to their absence in secreting lymphangiogenic inflammatory cytokines [138]. These findings strongly hinted at the correlation between lymphangiogenesis and aneurysm. First, lymphangiogenesis actively participated in the pathological process of aneurysm and the neoangiogenesis process is drived by inflammation. Second, the newly formed lymphatic vessels possibly transdifferentiated from PROX-1-expressing micro blood vessels. However, there are few studies that have investigated the influence of interventing in the process of lymphangiogenesis, and it remains unknown whether newly formed lymphatic vasculature is a friend or foe and whether it functions the same during different stages of disease development.

In conclusion, these findings provide insights to reveal the potential for lymphatic vessels to be a therapeutic target for aneurysm. However, the understanding we have established so far is not exhaustive enough, and further investigations are needed to improve the lesion stabilization and disease prognosis.

### 5.2. Atherosclerosis

Atherosclerosis is a multifocal and accumulated immune-inflammatory disease that is closely related to lipids, which usually occurs in medium and large elastic arteries [142]. Hyperlipidemia and lipid oxidation are considered as the key cause of atherosclerosis [130]. Its pathological features include lipid accumulation, fibrous tissue hyperplasia, and calcium salt deposition in the intima, which may eventually lead to plaque rupture, intra plaque hemorrhage, and thrombosis, causing a series of clinical consequences [131,132]. It is characteristic that the area of intensive lymphatic hyperplasia could usually detect an abundance of calcium and cholesterol crystals with few infiltrated cells [143]. Naturally, the relationship between the function of lymphatic vessels in reverse cholesterol transportation and the accumulation of cholesterol on the artery has been noticed by many researchers [144,145]. However, even though the existence of lymphatic vessels in atherosclerosis lesions is widely acknowledged, how lymphatic vasculature generation participates in the pathogenesis of the disease has not been thoroughly studied.

Adventitia on the vascular wall plays a vital role in maintaining vascular homeostasis. There, not only are lymphatic vessels scattered but also a large number of macrophages, T cells, dendritic cells, and a small number of B cells reside [146]. In progressive coronary atherosclerotic lesions, the number of lymphatic vessels in both intima and media increased in the physiological condition; however, lymphatics in adventitia did not increase obviously until the later course [143,147]. The density of adventitial lymphangiogenesis is positively correlated with the severity of atherosclerosis, which has already been found in mice and humans [148]. Lymphatic vessels at sites of atherosclerosis plaques derive from the adventitia of arteries adjacent to small blood vessels, which may be promoted by lymphokines secreted by inflammatory cells [149]. An early lymphatic drainage of local inflammatory cells that seems mainly attributed to T cells conferred a protection to dampening local immune response [147]. However, there are few reports that have declared the existence of lymphatic vessels in the plaque area, and it remains unknown whether lymphatics could stabilize the plaque or transport the fragments and lipids away from lesions.

To investigate whether lymphangiogenesis on different levels could influence the pathological process of atherosclerosis, researchers induced a lymphangiogenesis response by using recombinant VEGFC-156S, which improved the lymphatic molecular transport and inflammatory cell migration in an earlier stage [150,151]. Surprisingly, those stimuli or inhibition to VEGF-C/D-VEGFR3 pathway did not abrogate the lymphatic vasculature generation at adventitia plaque, indicating the significance of the non-classical lymphangiogenic backup pathways, for instance, the CXCL12/CXCR4 axis [147,152]. By injecting ApoA-I, the main protein constituent of plasma high-density lipoprotein, lymphatic transportation could be improved by strengthening the connection between lymphatic endothelial cells as well as diminishing the leakage from collecting lymphatic vessels, therefore reducing the accumulation of local aortic lipids [153]. In conclusion, these findings above may deliver a message that lymphatics are a potential therapeutic target. Contrary to common sense, the inhibition of lymphangiogenesis may also become an innovative method to stimulate the lymphatic drainage of atherosclerosis and induce plaque regression.

### 5.3. Transplant Arteriosclerosis

Lymphangiogenesis has been identified in various diseases, among which organ transplantation is special and has the most meaningful research value for the following reasons. First, perturbed lymphatic drainage and resultant lymphedema always emerge at an early stage due to a lack of surgical lymphatic anastomosis in organ transplantation [67]. Second, researchers noticed that the post-transplantation prognosis including fibrosis and patency was strongly relevant to the density of lymphangiogenesis in grafts [154,155]. Finally, tertiary lymphoid organs, which were recurrently identified in chronically rejected allografts, functioned as the powerhouse of local immunity and had a close relationship with lymphangiogenesis [156].

For a reference, inducing lymphangiogenesis by VEGF-C treatment improves the clearance of hyaluronan fragment from the lung allografts, which benefits the lung transplantation in a long-term effect [157]. However, in a study with kidney transplantation [158], authors found that the increased lymphangiogenesis by the overexpression of VEGF-C did not alleviate rejection but aggravated the neointimal formation and adventitial fibrosis. Close to kidney transplantation, inhibiting lymphanigogenesis produces some beneficial effects on cardiac allografts [90,91]. Acknowledging transplant arteriosclerosis as a chief limitation to the long-term survivals of patients with heart transplantation, we used a murine model of vascular allografts and demonstrated the abundant presence of tertiary lymphoid organs and lymphangiogenesis within grafted arteries [159]. Based on this discovery, we conducted the first comprehensive investigation and elaborated on the origin, characteristics, and regulating mechanisms of lymphangiogenesis in grafted vessels [160]. Most critically, we confirmed the early inhibition of lymphangiogenesis as a promising immunosuppressive target in transplant arteriosclerosis. Among the results, several key points provided direction and possibilities for future research about lymphatic progenitor cells in organ transplantation. 

**Figure 4:**
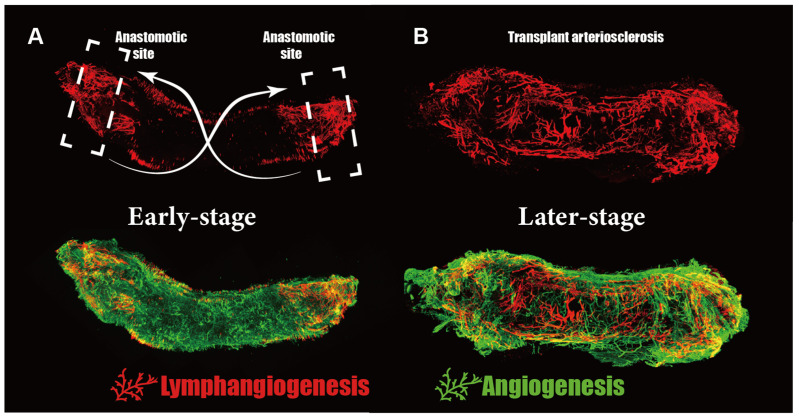
Detection of lymphangiogenesis by whole-mount staining LYVE1 (red) and CD31 (green) at different time points (2/4 week) after vascular transplantation. (**A**) The lymphangiogenesis visibly occurred at anastomotic sites and gradually spread towards the central part. (**B**) Until the fourth week post-transplantation, lymphatic vessels had already spread throughout the allograft vessels. Interestingly, in both time points, parts close to the anastomotic sites possessed the highest density and largest lumen area of lymphatics.

Initially, we employed LYVE-1 and CD31 markers for whole-mount staining and used a three-dimensional reconstruction technique to observe the growth patterns of newly formed lymphatic vessels and vasa vasorum [160]. The lymphangiogenesis and angiogenesis both visibly occurred at anastomotic sites and gradually spread towards the central part. More importantly, the prior appearance of angiogenesis also created opportunities for circulating lymphatic progenitor cells to be transported into the area where lymphangiogenesis initiated (Figure 4A,B).

Next, we took the advantage of the scRNA-seq technology and found out that there were three distinct LEC subsets in grafted arteries, designated as initial LECs, Foxp2hi, and Icam1hi-collecting LECs. Their gene expression profiles and functional enrichment were quite different, suggesting a complicated mature process and constant adaptation to the changing disease microenvironment. Although initial LECs tended to converge into collecting LECs and operate as a foundation of the lymphatic network within grafted vessels [160], whether lymphatic progenitor cells could differentiate into initial LECs and participate into the intact lymphatic network remains unknown. In fact, the extra and specific markers for lymphatic progenitor cells should be defined to improve the existing simple lineage-tracing strategies.

Although we did not find strong evidence about the incorporation of lymphatic progenitor cells into the lymphangiogenesis within allograft vessels, the possibility of circulating lymphatic progenitor cells constantly providing essential sources for “pre-existing lymphatic vessels”, which we believed were the major sources for emerging lymphatic vessels in grafted arteries, could not be ruled out [161]. The resolution has to be acquired by observation for a longer duration and the self-renew or extraneous-source proportion in lymphatic vasculature regeneration must be accurately evaluated.

**Figure 5:**
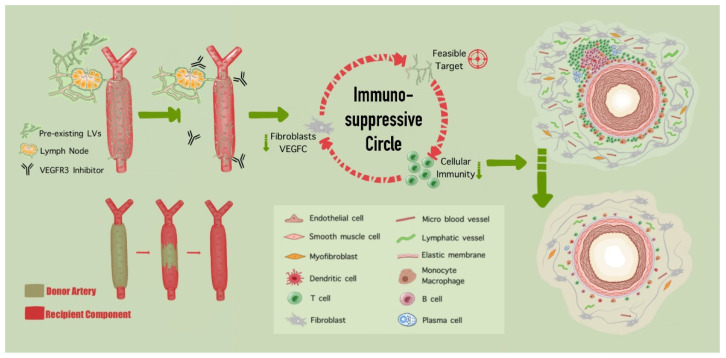
The impact of lymphangiogenesis in transplant arteriosclerosis. After vascular transplantation, the immediate recognition of donor artery components progressively induces immunological rejection response. After injecting a highly selective inhibitor for VEGFR3, the inhibition of lymphangiogenesis effectively suppresses the cellular adaptive immunity, and further ameliorates neointimal hyperplasia, adventitial fibrosis, and teritary lymphoid organs’ (TLOs) formation in vascular allografts. Resultantly, an immunosuppressive circle was reasonably achieved by the negative lymphangiogenesis manipulating.

To explore the possible therapeutic effects, we simultaneously evaluated the effects of stimulating or inhibiting lymphangiogenesis on vascular allografts [160]. Concentrating on the key regulating factor VEGF-C, we used adeno-associated virus encoding VEGF-C vectors (AAV9.VEGF-C) and concluded that the overexpression of VEGF-C could increase lymphanigogenesis and detrimentally exacerbate the transplant-related vascular remodeling. Conversely, by injecting a highly selective inhibitor for VEGFR3, the inhibition of lymphangiogenesis effectively ameliorated neointimal hyperplasia, adventitial fibrosis, and tertiary lymphoid organ formation in vascular allografts. In addition, we noticed that a progressive replacement by recipient components started to displace donor arteries immediately after vascular transplantation. As a result, inhibiting lymphangiogenesis in the early stage could alleviate neointimal formation in long-term prognosis, indicating that lymphangiogenesis could be the potential therapeutic target in transplant atherosclerosis treatment (Figure 5).

## 6. Conclusions and Perspectives 

Starting with a unitary concept that lymphangiogenesis has a solely venous origin, increasing evidence has uncovered the diversity and plasticity of lymphatic progenitor cells in both the embryonic and postnatal stages. In this review, we summarize the main sources for stem/progenitor cells in lymphvasculogenesis or lymphangiogenesis, mainly involved in the biological genesis and disease development. Although the differentiation from progenitor cells to mature LECs has been substantiated by many studies, the precise markers for lymphatic progenitor cells are still lacking. Referring to the heterogenic origin of cardiac lymphatics [10], the anatomical participation does not always represent the real cellular transformation. Due to the low frequency of endogenous stem cells and the complexity of definition, the current recognition of lymphatic progenitor cells remains very limited. Therefore, using advanced techniques, such as in-depth single-cell RNA sequencing or dual recombinase lineage tracing to analyze the lymphatic progenitor cell destiny and find related signaling pathways among different organs and diseases becomes the future research direction [162]. Moreover, because of the multidirectional differentiation potential, the favorable therapeutic application of lymphatic progenitor cells to increase the density of lymphangiogenesis remains controversial. Taken together, probing possible mechanisms for lymphatic progenitor cells may not only explain their differentiation, but also essentially guide the intentional induction for getting more benefits from their regenerative properties and ensure the treatment effectiveness. Specifically, in view of the above research, we concluded that targeting lymphangiogenesis may be a double-edged sword for the alleviation of vascular disease and the effective manipulating direction depends on the specific vascular pathogenesis. In general, because the function of lymphangiogenesis is specific to different organs, tissues, and diseases, individual-based treatment about whether to stimulate or inhibit the lymphangiogenesis could be a more flexible therapeutic choice for vascular diseases.

## Figures and Tables

**Table 1:** Recent findings about tissue-specific lymphatic progenitor cells.

Postnatal Organs	Species	Source	Differentiation	Reference
Eye	Mouse	(Cornea) CD11b^+^ macrophage; (Cornea) CD34^+^/VEGFR-3^+^ BM-LECP; (Choroid and retina) LYVE-1^+^ macrophages;	LEC	Maruyama, K., et al. [14] Religa, P., et al. [81] Xu, H., et al. [82]
Kidney transplant	Human	CD14^+^ VEGFR-3^+^ CD31^+^ VEGFR-2^−^ monocytes;	LEC	Kerjaschki, D., et al. [68]
Myocardial infarction	Mouse	Perivascular PDGFRβ^+^ PROX-1^+^ Podoplanin^+^ cells;	LEC, fibroblast	Cimini, M., et al. [69]
	Rat	BM-derived CD34^+^ VEGFR-3^+^ PROX1^+^ mononuclear cell;	LEC	Zhang, H.F., et al. [74]
Lewis lung carcinoma	Mouse	Native myeloid cells CD31^+^ PROX-1^+^;	LEC	Gordon, E.J., et al. [50]
Schlemm’s canal	Mouse, zebrafish, and human	Tie2^+^ venous EC progenitors;	LEC, EC	Kizhatil, K., et al. [55] Aspelund, A., et al. [54]
Melanoma	Mouse	Podoplanin^+^ BM-derived cells;	LEC	Lee, J.Y., et al. [83]
Breast cancer tissue	Human	M-LECPs co-expressed high levels of PROX1, LYVE-1, podoplanin, and VEGFR-3 and TIE-2^+^ monocytes;	LEC	Volk-Draper, L., et al. [84] Bron, S., et al. [85]
Diabetic retinopathy	Human	Ki-67^+^/CD117^+^VEGFR-3^+^ in CD31-positive vessels; Macrophage-derived LEC precursors CD68^+^ CD11b^+^ Prox1^+^ Lyve1^+^ VEGFR-3^+^ podoplanin^+^;	LEC	Loukovaara, S., et al. [86] Gucciardo, E., et al. [87]
Peritoneum	Mouse	Native macrophages CD11b^+^ F4/80^+^ LYVE-1^+^;	LEC	Hall, K.L., et al. [88]
Prostate cancer	Mouse	BM cells Podoplanin^+^ Lyve-1^+^ Prox1^+^;	LEC	Zumsteg, A., et al. [89]
Skin and ear wound	Mouse	Cultured Podopanin^+^ BM-MNC;	LEC	Lee, J.Y., et al. [83]
Insulinoma	Mouse	CD11b^+^ Myeloid cells (Prox1^+^, LYVE-1^+^, or Podoplanin^+^);	LEC	Zumsteg, A., et al. [89]

## Data Availability

Not applicable.
